# Validity of measurement of femoral anteversion angle using FEMORA® software based on two-dimensional radiographic imaging examination femur in children with cerebral palsy in Indonesia

**DOI:** 10.1016/j.heliyon.2023.e22243

**Published:** 2023-11-11

**Authors:** Tri Wahyu Martanto, Yusuf Rizal, Irwanto, Sulis Bayu Sentono, Rosy Setiawati, Sri Andreani Utomo, Prastiya Indra Gunawan, Nurul Kusuma Wardani, Prima Hari Nastiti, Rachmat Agung Widodo, Moon Seok Park, Arif Zulkarnain, Hizbillah Yazid, Hendra Cahaya Kumara, Muhammad Ihsan Kitta

**Affiliations:** a Department of Orthopaedic and Traumatology, Faculty of Medicine, Universitas Airlangga/ Dr. Soetomo General Academic Hospital, Surabaya 60286, Indonesia; b Department of Child Health, Faculty of Medicine, Universitas Airlangga/ Dr. Soetomo General Academic Hospital, Surabaya 60131, Indonesia; c Department of Radiology, Faculty of Medicine, Universitas Airlangga/ Dr. Soetomo General Academic Hospital, Surabaya 60131, Indonesia; d Department of Physical Medicine and Rehabilitation, Faculty of Medicine, Universitas Airlangga/ Dr. Soetomo General Academic Hospital, Surabaya 60131, Indonesia; e Department of Orthopaedic Surgery, Seoul National University College of Medicine/Seoul National University Bundang Hospital, Sungnam, Gyeonggi, South Korea; f Department of Orthopaedic and Traumatology, Prof. Dr. R. Soeharso Orthopaedic Hospital, Sebelas Maret University, Surakarta 57126, Indonesia; g Department of Orthopaedic Surgery, Muhammadiyah University of Makassar, Makassar 90221, Indonesia

**Keywords:** CT scan, X-ray, Femoral anteversion angle, Cerebral palsy

## Abstract

**Introduction:**

Children with spastic cerebral palsy (CP) often show an increase in femoral anteversion angle (FAA). Computed tomography (CT) scan is the main modality for evaluating FAA in these patients, however, due to significant radiation exposure, it carries a high carcinogenic risk. FEMORA® software is expected to be able to accurately assess FAA even with conventional X-ray images that only require low radiation exposure. However, its validity has not been tested in various populations or CT devices. This study aimed to validate the FEMORA® software by comparing it to CT scans done on an Indonesian population.

**Material and methods:**

All spastic CP patients of the outpatient clinic at Dr. Soetomo Hospital between March and November 2022, were included. The FEMORA® Software evaluation was performed by three examiners. The calculation results were averaged and compared with those of the CT scan. Intraclass correlation coefficient (ICC), reliability, and correlation were be assessed.

**Results:**

There were 36 patients included in this study. Most were female (n = 22; 61,1 %) and the average age was 7,28 years old. Interobserver preoperative analysis using ICC showed good outcomes (p = 0.918; 95 % CI, 0.858–0.955). FAA measurement results using FEMORA® and CT scans were 41,71 ± 12,90 and 32,68 ± 11,85, respectively. Correlation coefficient between the two values is 0.634 (p < 0.001).

**Conclusion:**

FEMORA® software demonstrates a good and significant correlation with FAA measurement using CT scan.

## 1 Introduction

The femoral anteversion angle (FAA) is the angle formed between the axis of the femoral neck and the axis of the coronal plane of the femoral condyle (condylar plane) [1,2]. An increase in FAA will decrease the arm abduction moment of the hip abductor and lead to a cosmetically poor gait pattern [3], inefficient gait, and functional limitations [[4], [5], [6], [7]].

In measuring FAA, a physician may do physical examination, conventional radiography, or computed tomography (CT) scans. Clinical examination using the Trochanteric Prominence Angle Test is reliable for measuring FAA and is a useful screening tool. The advantage of this examination is that it can be performed repeatedly, is inexpensive, safe and does not involve radiation exposure [8,9]. However, this method is less accurate and reliable due to muscle spasticity, deviation in the position of the femur and deformity of the bones [10].

Conventional 2D radiography is generally used for the diagnosis and follow-up of the lower extremities, but it has the disadvantage of being sensitive to the orientation of the patient and bone deformities. A CT scan overcomes this disadvantage and provides more accurate calculations [[11], [12], [13]]. By being able to scan through multiple slices and even create a 3D reconstruction of the affected site, CT is considered the “gold standard” imaging technique for evaluating FAA [2,14] and is a reliable and valid method [10]. The disadvantage of a CT scan is that it has a high carcinogenic risk due to the high radiation exposure, especially in the pediatric population [15].

FEMORA**®** Software developed by Didim Co., Ltd. tries to solve the radiation and cost problem of CT scans. By using only biplanar X-rays and the help of FEMORA**®** software, 3D images can be reconstructed and the need for a CT scan is reduced [16]. If validity and reliability are proven, it will help reduce the burdens of cost and radiation exposure in patients. Consequently, this program may replace the need for CT scans or as reference standards for measuring FAA.

To prove the validity and reliability of this software before use as a standard for measuring FAA in a clinical setting, it is important for the software to pass repeated tests and carry out in research centers and different populations [17,18]. In assessing the capacity of this software to measure FAA, patients with highly variant FAA are needed.

Cerebral palsy (CP) is a chronic condition with considerable impact on affected individuals. Children with CP suffer from motor problems, frequent seizure/epilepsy, and other disorders [19,20]. Such chronic disorder combined with immature femur results in changes on FAA angle [10]Thus, patients with spastic CP tend to have varied FAA and will be ideal candidates for assessing this software.

As of the conduction of this study, the validity and reliability testing of the FEMORA® software has been performed in only one study [16]. Therefore, this study was conducted to evaluate and re-validate the application of FEMORA® medical 3D image software measurement by focusing on FAA measurements.

## 2 Methods

This is an observational analytic study with a cross-sectional approach to evaluate the validity of using plain radiographs of the anteroposterior and lateral femur calculated using the FEMORA® Software to assess FAA in patients with CP 2–12 years old who attended the outpatient clinic at Dr. Soetomo General Hospital from March 2022 to January 2023. Informed consent was obtained from each participant and ethical approval was obtained from the Ethics Committee of the Dr. Soetomo General Hospital Surabaya.

The criteria in this study were: (1) patients with Spastic CP willing to undergo pelvic radiography, femur radiography, and CT scan, (2) patients who are not currently being treated for other diseases, (3) adequate radiographic coverage or quality, (4) no femur fracture, hip joint contracture, or hip joint dislocation, and (5) patients had never had implants.

**Fig. 1:**
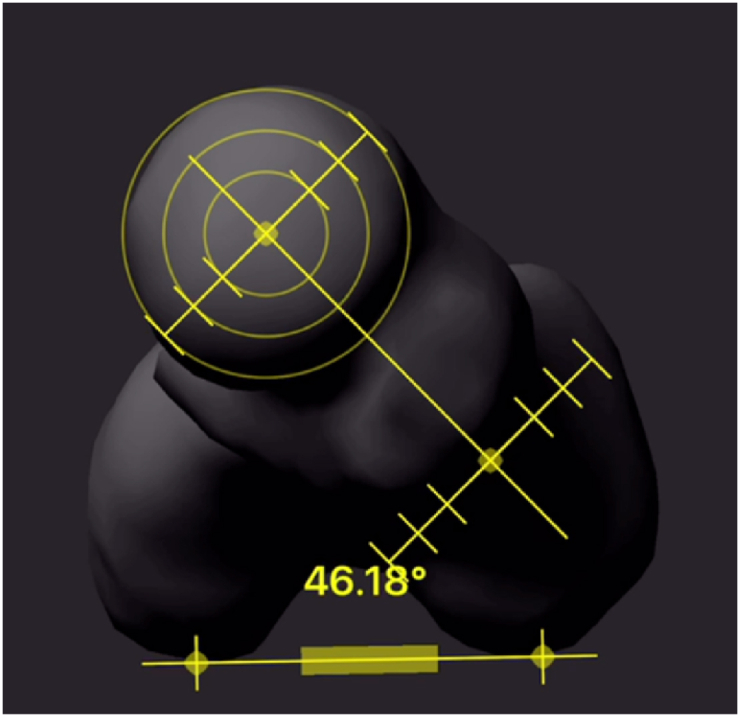
Sample image of the 3D reconstruction from two plane X-rays and the angle measurement using FEMORA® software.

**Fig. 2:**
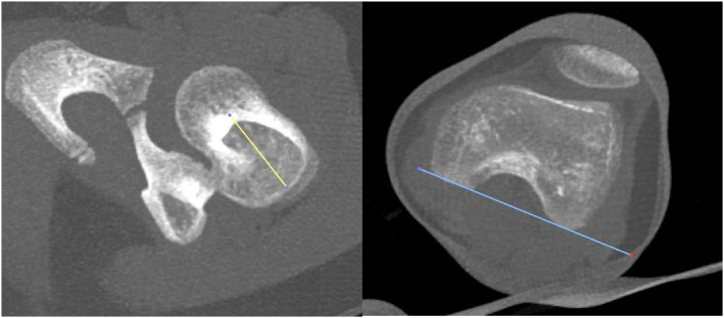
Sample image of the measured CT scan. The measurement method is as described by Hernandez et al. [12]. On the left is the angle taken from the femur neck. On the right is the angle taken from the posterior border of the medial and lateral condyle. The measured angle is the difference between the two lines.

FAA was assessed using clinical examination [7], femur radiography [21], FEMORA® software, and CT scans [12] of the patient femur. A sample picture of the measurement using Femora® and CT scan are shown in Fig. 1, Fig. 2, respectively.

To obtain FAA from FEMORA® software we first obtained conventional radiograph from the patient femur. We obtain anteroposterior and lateral images. Then, software application is embedded in ipad and the camera took images of both radiographs. The images the application was developed to provide not only automatic contouring with a graph-cut algorithm but also an intuitive touch interface for modifying the contour of a radiograph and navigating the 3D view to verify the reconstruction result [16]. Then, the software will calculate the FAA angle automatically. The test was repeated three times for each femur.

Validity and reliability were determined by three examiners (T.W.M., H·C·K., and M.I·K.). The appraiser was not involved in the development of the software.

After the three examiners conducted their assessment, interobserver reliability was assessed visually and quantitatively. Visually, an analysis was carried out using the Bland-Altman test to compare the results of the assessments between examiners 1 and 2, examiners 2 and 3, and examiners 1 and 3. Quantitatively, the intraclass correlation coefficient (ICC) of the three examiners was calculated.

ICC and 95 % confidence interval (CI) are used to infer interobserver reliability and was calculated using a 2-way random effects model assuming absolute agreement. An ICC of 1 indicates perfect reliability and an ICC of >0.8 indicates excellent reliability [22]. Pearson correlation coefficients were used to determine the validity of the measurement of FAA with use of the FEMORA® software. The Pearson correlation coefficient was characterized as poor (0.00–0.2), fair (0.21–0.4), moderate (0.41–0.6), good (0.61–0.8), or excellent (0.81–1.00) [22]. The Bland-Altman comparison was performed to assess the validity of the assessment using FEMORA® software visually based on a scattered plot [23].

Statistical analysis was performed using SPSS software for Windows (version 25.0; IBM), and the null hypothesis of no difference was rejected if the p-value was <0.05.

## 3 Results

Overall, a total of 36 patients participated in this study. The evaluation results and sample demographic data are tabulated in Table 1. Most patients were female (n = 22; 61,1 %) and the mean age was 7,28 years with a range of 3–12 years.

**Table 1:** Patient demographics and measurements.

Description	N	Value	Range
Gender (Males)	14	14/36 (38,9 %)	
Age (Years)		7,17 ± 2,24	3–12
Measurement Using Physical Examination	36	26,85 ± 6,26	14–48
Measurement Using Conventional Radiograph	36	45,09 ± 17,97	14,9–83,2
Measurement Using CT Scan 3D	36	32,68 ± 11,85	7,95–65,3
Measurement Using FEMORA® Software	36	41,71 ± 12,90	18,3–72,1

An interobserver reliability test was conducted to determine whether the results of the analysis of the three examiners were consistent and free of bias. This test was carried out visually using the Bland-Altman chart and quantitatively using the ICC.

**Fig. 3:**
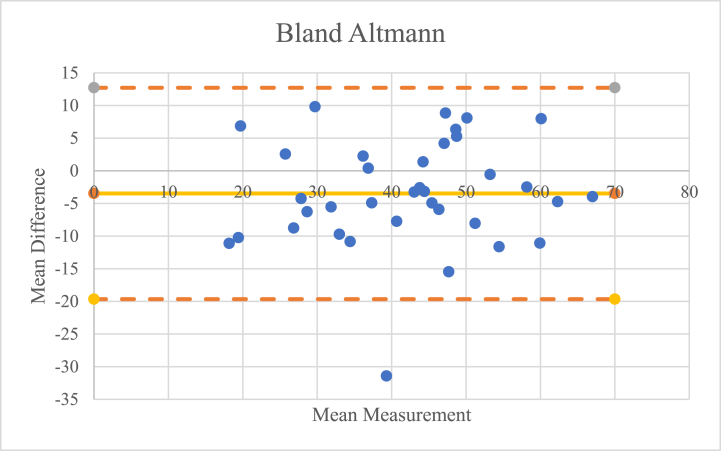
Bland-Altmann chart comparison between the 1st and 2nd examiners.

**Fig. 4:**
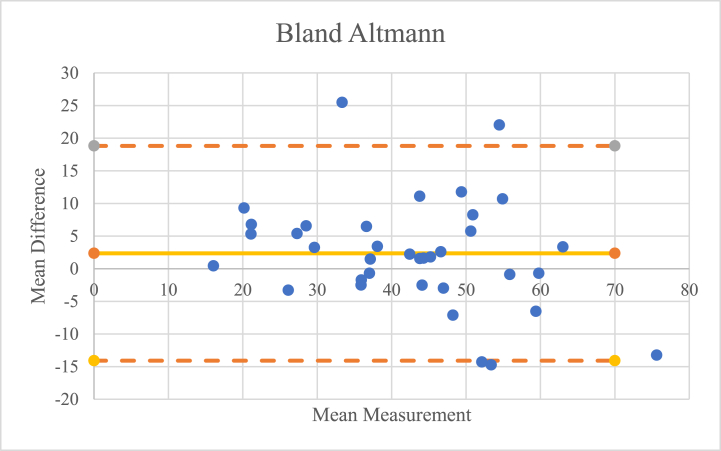
Comparison of the Bland-Altmann chart between the 2nd and 3rd examiners.

**Fig. 5:**
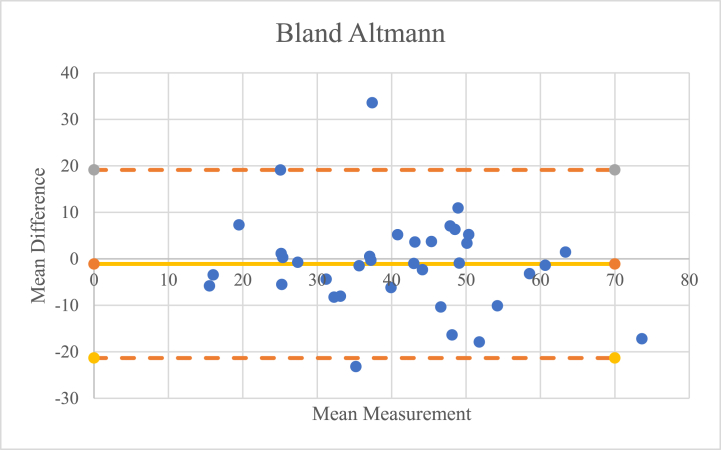
Bland-Altmann chart comparison between the 1st and 3rd examiners.

Visually, from the Bland-Altman chart, as shown in Fig. 3, Fig. 4, Fig. 5, it was found that only three of the 36 data points were outside the reasonable range, so it can be concluded that the risk of bias in the assessment is minimal.

The ICC analysis showed excellent correlation between examiners (ICC, 0.918; 95 % CI, 0.858–0.955) which indicated that the results of the FAA angle assessment using X-ray processed with the FEMORA® software were consistent across multiple examiners. In future use in the field, measurements by several trained experts will not show a significant difference.

**Fig. 6:**
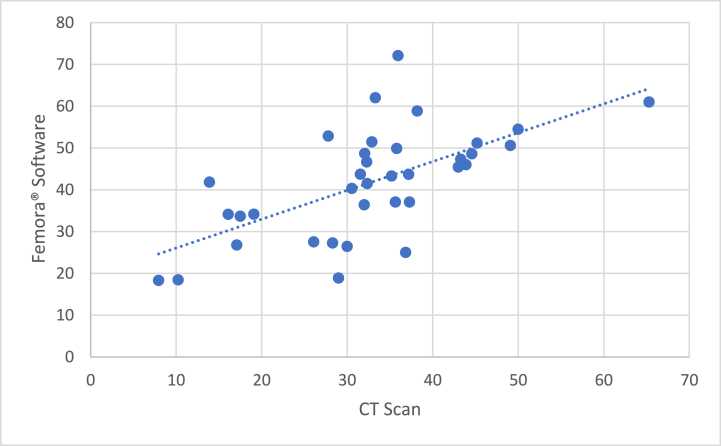
Scatter plot showing the correlation between CT scans and FEMORA® software.

The measurement results from the CT scan and FEMORA® software were compared using Pearson's correlation test which found a significant and good correlation (r = 0.634; p < 0.001). From The scatter plot (Fig. 6), It can be observed that the point of association between these two assessment methods showed a clear trend. This shows that the relationship between these two measurement methods is fairly strong.

## 4 Discussion

FEMORA® is a newly developed software that can reconstruct 3D images of the femur from conventional radiographs. In previous studies, this application has shown convergence validity and good interobserver reliability [16]. This study aims to review these results to determine whether validity and reliability also apply to the study population in different centers and geographic locations.

FEMORA® is made to reduce radiation exposure in patients who require routine checkup with CT scans, such as patients with CP. Several notable advantages of FEMORA® compared to CT scans are, firstly, the app only requires a mobile device and a conventional radiograph to visualize a 3D image of the femur. Second, the FEMORA® software could cover a wide FAA: from 18° to 83°, and thus it can be used for various cases. Third, FEMORA® does not require the patient to remain still for long time periods like on CT scans, making it even more convenient for assessing children who are usually less cooperative.

The mobile application FEMORA® has similar research by Sung et al. They found similarly excellent interobserver reliability (ICC, 0.953; 95 % CI, 0.917–0.975). Visual analysis using Bland-Altman plots was also similarly excellent, with only a few points outside the upper/lower limit. Validity is also similar but the correlation by Sung et al. was higher score at 0.968 [16]. Younger patients might have contributed to the lower correlation in our study. Measurement of angles in younger patients are usually more prone to errors due to smaller bone diameters and more non-ossified cartilage in the bones [24].

An alternative method that can be used is a biplanar X-ray which is interpreted using a specific system/program. One program that most closely resembles FEMORA® is EOS imaging. EOS was originally used for 3D reconstruction of the spine, but has been found to have good validity and reliability for the measurement of femoral anteversion angle [25,26]. The radiation dose of the EOS imaging system has been reported to be much less than that of a CT scan. Folinais et al. showed that the mean radiation dose from the EOS system was 0.18 mGy for the AP view and 0.45 mGy for the LAT view, and from a CT scan it was 8.4–15.6 mGy [27]. In addition, Deschenes et al. demonstrated that full spinal EOS imaging yields 6 to 9 times less radiation than conventional radiography [28]. This huge difference in radiation dose would be similar as in FEMORA®. However, the EOS system is not suitable for use in some hospitals and countries due to its high cost, the need for specialized equipment, and space limitations.

The limitations of this study are the limited number of patients and the lack of repeated CT scan measurement. More patients included in this study would certainly increases the validity of this study. Moreover, CT scan result is also subjective because the measurement is done by single radiologist. Therefore, the data can be false. Repeated CT scan measurement by different assessor will increase the validity of the CT scan measurement.

## 5 Conclusion

The FAA of the samples measured using FEMORA® software and CT scan was, consequently, 41,71 ± 12,90 and 32,68 ± 11,85. We also found excellent reliability (p = 0.918; 95 % CI, 0.858–0.955) and a good correlation with the CT scan results (r = 0.634; p < 0.001). Thus, FEMORA is a good alternative to CT scans as it shows a good correlation and reliability and reduces patient radiation exposure.

## Ethical approval

Approval for this study was obtained from our ethic and medico-legal committee of Dr Soetomo Hospital, Surabaya, Indonesia (Reference number: 2009/KEPK/VI/2020).

## Funding sources

This work was supported by 10.13039/501100008463Universitas Airlangga, Indonesia, award number 419/UN3.14/PT/2020.

## Data availability statement

The data will be made available upon request. Contact the corresponding author to retrieve the data.

## Additional information

No additional information is available for this paper.

## CRediT authorship contribution statement

**Tri Wahyu Martanto:** Conceptualization, Investigation, Methodology, Supervision, Validation. **Yusuf Rizal:** Data curation, Formal analysis, Project administration, Writing – original draft, Writing – review & editing. **Irwanto:** Conceptualization, Methodology, Supervision, Conceptualization, Methodology, Supervision. **Sulis Bayu Sentono:** Conceptualization, Funding acquisition, Investigation, Methodology. **Rosy Setiawati:** Data curation, Investigation, Resources, Software. **Sri Andreani Utomo:** Data curation, Investigation, Resources, Software. **Prastiya Indra Gunawan:** Resources. **Nurul Kusuma Wardani:** Project administration, Resources. **Prima Hari Nastiti:** Formal analysis, Funding acquisition, Project administration. **Rachmat Agung Widodo:** Data curation, Formal analysis, Funding acquisition, Project administration. **Moon Seok Park:** Conceptualization, Methodology, Software, Supervision. **Arif Zulkarnain:** Visualization, Writing – original draft, Writing – review & editing. **Hizbillah Yazid:** Visualization, Writing – original draft, Writing – review & editing. **Hendra Cahaya Kumara:** Investigation, Validation. **Muhammad Ihsan Kitta:** Investigation, Validation.

## Declaration of competing interest

The authors declare that they have no known competing financial interests or personal relationships that could have appeared to influence the work reported in this paper.

## References

[1] Lee D.Y., Lee C.K., Cho T.J. (1992). A new method for measurement of femoral anteversion. – a comparative study with other radiographic methods. Int. Orthop..

[2] Mesgarzadeh M., Revesz G., Bonakdarpour A. (1987). Femoral neck torsion angle measurement by computed tomography. J. Comput. Assist. Tomogr..

[3] Sung K.H., Kwon S.S., Chung C.Y., Lee K.M., Cho G.H., Park M.S. (2018). Long-term outcomes over 10 years after femoral derotation osteotomy in ambulatory children with cerebral palsy. Gait Posture.

[4] Pirpiris M., Trivett A., Baker R., Rodda J., Nattrass G.R., Graham H.K. (2003). Femoral derotation osteotomy in spastic diplegia. Proximal or distal?. J Bone Joint Surg Br.

[5] Kelly D., Spence D. (2015).

[6] Lee E.H., Nather A., Goh J., Teng B., Bose K. (1985). Gait analysis in cerebral palsy. Ann. Acad. Med. Singapore.

[7] Rosenbloom L. (1988). Orthopaedic management in cerebral palsy. Arch. Dis. Child..

[8] Tamari K., Tinley P., Briffa K., Breidahl W. (2005). Validity and reliability of existing and modified clinical methods of measuring femoral and tibiofibular torsion in healthy subjects: use of different reference axes may improve reliability. Clin. Anat..

[9] Guenther K.P., Tomczak R., Kessler S., Pfeiffer T., Puhl W. (1995). Measurement of femoral anteversion by magnetic resonance imaging - evaluation of a new technique in children and adolescents. Eur. J. Radiol..

[10] Chung C.Y., Lee K.M., Park M.S., Lee S.H., Choi I.H., Cho T.J. (2010). Validity and reliability of measuring femoral anteversion and neck-shaft angle in patients with cerebral palsy. J Bone Joint Surg Am.

[11] Seber S., Hazer B., Köse N., Göktürk E., Günal I., Turgut A. (2000). Rotational profile of the lower extremity and foot progression angle: computerized tomographic examination of 50 male adults. Arch. Orthop. Trauma Surg..

[12] Hernandez R.J., Tachdjian M.O., Poznanski A.K., Dias L.S. (1981). CT determination of femoral torsion. AJR Am. J. Roentgenol..

[13] Murphy S.B., Simon S.R., Kijewski P.K., Wilkinson R.H., Griscom N.T. (1987). Femoral anteversion [internet]. J. Bone Joint Surg..

[14] Thépaut M., Brochard S., Leboucher J., Lempereur M., Stindel E., Tissot V. (2016). Measuring physiological and pathological femoral anteversion using a biplanar low-dose X-ray system: validity, reliability, and discriminative ability in cerebral palsy. Skeletal Radiol..

[15] Brenner D.J., Hall E.J. (2007). Computed tomography — an increasing source of radiation exposure. N. Engl. J. Med..

[16] Sung K.H., Youn K., Chung C.Y., Kitta M.I., Kumara H.C., Min J.J. (2020). Development and validation of a mobile application for measuring femoral anteversion in patients with cerebral palsy. J. Pediatr. Orthop..

[17] Abd Elmagid D.S., Magdy H. (2021). Evaluation of risk factors for cerebral palsy. Egypt J Neurol Psychiatry Neurosurg.

[18] Hale A.T., Akinnusotu O., He J., Wang J., Hibshman N., Shannon C.N. (2021). Genome-wide association study identifies genetic risk factors for spastic cerebral palsy. Neurosurgery.

[19] Prastiya I.G., Risky V.P., Mira I., Retno A.S., Darto S., Erny P. (2018). Risk factor of mortality in Indonesian children with cerebral palsy. J. Med. Invest..

[20] Yulianti R., Gunawan P.I., Saharso D. (2021). Comparison of clinical characteristics and neuroimaging of cerebral palsy with and without epilepsy in children. Indian J Med Forensic Med Toxicol.

[21] Ogata K., Goldsand E.M. (1979). A simple biplanar method of measuring femoral anteversion and neck-shaft angle [Internet]. J. Bone Joint Surg..

[22] Min J.J., Youn K., Oh S., Sung K.H., Lee K.M., Park M.S. (2022). Development and validation of a mobile application for measuring tibial torsion. J. Bone Jt. Surg. Am. Vol..

[23] Martin Bland J., Altman D. (1986). Statistical methods for assessing agreement between two methods of clinical measurement. Lancet.

[24] Tomczak R.J., Guenther K.P., Rieber A., Mergo P., Ros P.R., Brambs H.J. (1997). MR imaging measurement of the femoral antetorsional angle as a new technique: comparison with CT in children and adults. AJR Am. J. Roentgenol..

[25] Rosskopf A.B., Buck F.M., Pfirrmann C.W.A., Ramseier L.E. (2017). Femoral and tibial torsion measurements in children and adolescents: comparison of MRI and 3D models based on low-dose biplanar radiographs. Skeletal Radiol..

[26] Pomerantz M.L., Glaser D., Doan J., Kumar S., Edmonds E.W. (2015). Three-dimensional biplanar radiography as a new means of accessing femoral version: a comparative study of EOS three-dimensional radiography versus computed tomography. Skeletal Radiol..

[27] Folinais D., Thelen P., Delin C., Radier C., Catonne Y., Lazennec J.Y. (2013). Measuring femoral and rotational alignment: EOS system versus computed tomography. Orthop Traumatol Surg Res.

[28] Deschênes S., Charron G., Beaudoin G., Labelle H., Dubois J., Miron M.C. (2010). Diagnostic imaging of spinal deformities: reducing patients radiation dose with a new slot-scanning X-ray imager. Spine.

